# Development of a crocetin–glycyrrhizic acid supramolecular composite pigment for enhancing meat color and reducing contaminant formation during heat processin

**DOI:** 10.1016/j.fochx.2026.104188

**Published:** 2026-07-17

**Authors:** Qinxue Ni, Haici Lan, Shuang Chen, Xiaolong Zhang, Yanming Ren, Wenhao Xu, Youzuo Zhang

**Affiliations:** a Zhejiang Provincial Key Laboratory of Resources Protection and Innovation of Traditional Chinese Medicine, College of Food and Health, Zhejiang A & F University, Linan 311300, China; b Collaborative Innovation Center of Yangtze River Delta Region Green Pharmaceuticals, Zhejiang University of Technology, Hangzhou 310014, China

**Keywords:** Crocetin, Disodium glycyrrhizinate, Supramolecular pigment, Meat product application, Resonant acoustic mixing, Crocetin (PubChem CID: 5281232), Disodium glycyrrhizinate (PubChem CID: 118984432), Glycyrrhizic acid (PubChem CID: 14982), Crocin-I (PubChem CID: 5281233), 5-Hydroxymethylfurfural (PubChem CID: 237332), *N*-Nitrosodimethylamine (PubChem CID: 6124), 2,2-Diphenyl-1-picrylhydrazyl (PubChem CID: 2735032), 2,2′-Azino-bis(3-ethylbenzothiazoline-6-sulfonic acid) (PubChem CID: 5464076)

## Abstract

A supramolecular pigment (Croc-Na_2_GA) was developed by the self-assembly of crocetin and disodium glycyrrhizinate using resonant acoustic mixing (RAM) technology for meat-coloring applications. The resulting complex formed stable nanomicelles (197.30 nm) that exhibited enhanced solubility and robust stability across pH 8–13 and under thermal processing conditions. Molecular docking confirmed strong binding affinities with myofibrillar proteins, including myoglobin, actin, and myosin, thereby facilitating pigment retention within meat matrices. Application in chicken patties produced a stable golden-yellow hue with sensory attributes comparable to those of commercial gardenia yellow. Notably, Croc-Na_2_GA demonstrated potent antioxidant capacity and significantly reduced the formation of hazardous Maillard reaction byproducts, decreasing 5-HMF and NDMA levels by approximately 50% after reheating. This bio-based nanosystem provides a multifunctional, clean-label colorant for enhanced meat products.

## 1 Introduction

Thermally processed meat products constitute a major food category and play a central role in dining experiences worldwide (Harlina et al., 2025; Manzoor et al., 2021). These products typically undergo heat treatments such as steaming, smoking, or baking, which inactivate microorganisms, enhance flavor, and extend shelf life (Bhat et al., 2021). The color of meat products is a critical quality attribute that profoundly influences consumer acceptance (Tomasevic et al., 2021). However, intrinsic pigments in meat, mainly hemoglobin and myoglobin, can undergo denaturation during thermal processing, leading to undesirable color degradation (Han et al., 2024). Additionally, reactions between proteins and sugars at elevated temperatures, such as the Maillard reaction (Starowicz & Zieliński, 2019), and lipid peroxidation during storage (Flores et al., 2019; Wang et al., 2023) can result in undesirable brown or black discoloration in processed meat. These changes significantly affect the sensory attributes of the products (Dumandan et al., 2023; Schrenk et al., 2023). Consequently, food colorants and color-protection additives have become essential ingredients in the meat processing industry. Among these additives, warm yellow and orange-red pigments play a vital role in harmonizing with the brown hues typical of red meat and compensating for the paler appearance of white meat (Barido et al., 2022; Olas et al., 2021). In addition to improving visual appeal, these pigments contribute to a more appetizing appearance (Coultate & Blackburn, 2018). Warm yellow colorants are widely used in regional specialties such as curry and barbecue dishes (García-Casal et al., 2016) and thus remain among the most commonly employed colorants in meat product formulations.

Currently, the most frequently used yellow food colorants include sunset yellow, lemon yellow, red currant yellow, gardenia yellow, and curcumin (Bashir et al., 2024). The first two are synthetic dyes known for their excellent coloring performance and stability. However, they pose health risks due to the potential release of toxic substances during thermal processing (Amchova et al., 2024). In contrast, natural pigments such as gardenia yellow and curcumin are considered safer and more acceptable to consumers. Nonetheless, their thermal stability, resistance to discoloration, and tolerance to metal ions remain inferior to those of synthetic dyes (Alegbe & Uthman, 2024). These limitations hinder their application in meat processing, particularly under prolonged heat treatments such as stewing and roasting.

Given the growing demand for functional natural yellow pigments in the food industry, research on the stability and compatibility of natural colorants in meat systems has become increasingly important (Ke et al., 2023; Novais et al., 2022). First, numerous studies have focused on expanding the sources of natural yellow pigments for food applications. For instance, pigments derived from *Thermomyces* sp. (Poorniammal et al., 2015), oyster mushrooms (Zhang et al., 2022), *Penicillium brevicompactum* (Poorniammal et al., 2021), and *Opuntia ficus-indica* pulp (Carmona et al., 2021) have demonstrated good stability and coloring performance. However, these pigments still require comprehensive safety assessments before being widely applied in food (Thakre et al., 2023). Second, developing naturally derived yellow pigments into complex formulations that comply with food safety regulations has become a major research focus. For example, the thermal and light stability of gardenia yellow pigment (GYP) has been enhanced by microencapsulation using maltodextrin or protein-based wall materials (Jurić et al., 2022; Tang et al., 2022). Similarly, curcumin has been loaded onto pectin matrices to improve its solubility and permeability (Nguyen et al., 2014; Xie et al., 2021), while other investigations have explored the use of fucoidan, safflower yellow, and erythrulose (Lu et al., 2023). Although these physical encapsulation strategies effectively enhance pigment stability, they also increase particle size (Chan et al., 2023), potentially reducing coloring efficiency—particularly when hydrophilic carriers are used. These carriers are prone to fading under humid post-application conditions, leading to decoloration and packaging staining issues (Luo et al., 2022).

As a global culinary leader, China has developed sophisticated meat processing and coloration techniques grounded in centuries of empirical knowledge and traditional practices. The use of natural spices to preserve chromatic integrity and enhance flavor profiles in meat products remains deeply rooted in Chinese cuisine. Among these, gardenia (the fruit of *Gardenia jasminoides* Ellis) serves as a pivotal yellow colorant widely used in meat processing (Jin & Lee, 2024)*.* Notably, analyses of traditional Chinese meat-coloring formulations have revealed that more than 80% of yellow-toned brine recipes combine gardenia with licorice root *(Glycyrrhiza uralensis* Fisch) without the addition of baking soda (Lin, 2024). During thermal processing with this spice combination, the resulting meat products exhibit bright coloration, greater oxidation resistance, and improved color retention even after soaking. These traditional formulations provide valuable insights into designing efficient and stable composite colorants for modern meat processing applications.

By integrating traditional phytochemical knowledge with advanced nanofabrication techniques, this study investigates the compositional patterns and mechanisms of color development in traditional Chinese marinated meat recipes. The chemical transformations occurring during the marination of such braised meat products were analyzed, and, based on these insights, a supramolecular natural yellow pigment with enhanced coloring ability, stability, and permeability was synthesized. Subsequently, the application of this pigment in meat processing was evaluated, together with its capacity to inhibit the formation of carcinogenic compounds—including radicals generated from protein–lipid oxidation (Hadidi et al., 2022), nitrosamines (Deveci & Tek, 2024), and 5-hydroxymethylfurfural (5-HMF) (Noh et al., 2024)—during repeated reheating of meat products. Ultimately, this work aims to establish a multifunctional natural pigment for innovative clean-label meat products (Mcdonnell et al., 2013) while elucidating its potential mechanisms of action.

Compared with existing natural pigment encapsulation and modification technologies, the supramolecular strategy employed in this study offers several distinct technical advantages. Although microencapsulation using maltodextrin or protein-based wall materials effectively improves thermal stability, it often results in significantly increased particle sizes that compromise coloring efficiency within meat matrices. Similarly, curcumin-loaded pectin matrices and related carrier systems demonstrate improved solubility but exhibit limited stability under the acidic conditions commonly encountered in meat systems. More broadly, physical encapsulation strategies, while beneficial for enhancing pigment stability, generally increase particle size and may contribute to decoloration and packaging staining under humid conditions. In contrast, the Croc-Na₂GA supramolecular system integrates the functions of pigment, carrier, and stabilizer into a single nanoscale architecture, thereby enhancing tissue permeability without compromising color intensity. Furthermore, the resonant acoustic mixing (RAM) preparation method distinguishes this approach by enabling solvent-free and scalable solid-phase synthesis, thereby avoiding the environmental and safety concerns associated with conventional solvent-based encapsulation processes.

Collectively, these characteristics position the Croc-Na₂GA system as a multifunctional platform capable of simultaneously addressing the coloration, stability, and safety requirements of meat-processing applications. The core innovations of this study are threefold: (i) the integration of pigment, carrier, and stabilizer functions into a single nanoscale supramolecular architecture, enabling enhanced tissue permeability without compromising color intensity; (ii) the solvent-free and scalable preparation of this complex through RAM, distinguishing it from conventional solvent-based encapsulation approaches; and (iii) the simultaneous enhancement of coloration, stability, and safety in meat processing through a multifunctional platform inspired by traditional culinary knowledge.

## 2 Materials and methods

### 2.1 Materials

Gardenia, licorice root, and other spices were sourced from the Whole Flavor Edible Flavor Store (Sichuan, China). Pig skin, chicken leg, chicken breast, cod, and squid were purchased from Yonghui Supermarket Co., Ltd. (Hangzhou, China). All ingredients used in the preparation of processed meat products were of food-grade quality.

Crocetin (Croc), gardenia yellow, crocin-I, glycyrrhizic acid (GA), 5-hydroxymethylfurfural (5-HMF), *N*-nitrosodimethylamine (NDMA), and disodium glycyrrhizinate (Na_2_GA) were purchased from Yuanye Bio-Technology Co., Ltd. (Shanghai, China). Sodium carbonate (Na_2_CO_3_), sodium chloride, acetonitrile, methanol, 2,2-diphenyl-1-picrylhydrazyl (DPPH), 2,2′-azino-bis(3-ethylbenzothiazoline-6-sulfonic acid (ABTS), hydrogen peroxide, ferrous chloride, 1,10-phenanthroline, pyrogallol, and methanol were obtained from Chengle Technology Co., Ltd. (Hangzhou, China). All reagents were of analytical reagent (AR) or biochemical reagent (BR) grade purity.

### 2.2 Design and formulation of the Croc-Na₂GA supramolecular pigment based on traditional applications of gardenia

Deepseek-V3 (Hangzhou Deep Seeking Artificial Intelligence Basic Technology Research Co., Ltd., Hangzhou, China) was used to extract and collect representative marinated recipes for thermally processed meats from Chinese cultural heritage databases (including www.meishichina.com, www.xiachufang.com, www.cooks.tw, and www.themealdb.com). Common natural meat-coloring materials, namely gardenia (“栀子”), *Monascus* (“红曲”), *Lithospermum* (“紫草”), caramel (“焦糖/糖色/红糖”), and curcumin (“姜黄”), were identified and used as keywords for further searches. Recipes containing these colorants were collected and analyzed, and the frequency of spices appearing in the same recipe as each coloring material was quantified. The data were visualized using VOSviewer (version 1.6.20) (Wong, 2018) to generate a correlation heatmap illustrating the co-occurrence rates between the four colorants and other spices. Subsequently, recipes containing gardenia were further analyzed to assess the frequency of spices co-occurring with it. Spices that appeared in more than 2% of the collected recipes were selected for experimental validation.

Each selected spice was mixed with gardenia in a 1:1 (*w*/w) ratio, followed by the addition of a 1% (w/w) Na_2_CO_3_ solution. The mixture was soaked for 30 min and then co-boiled at 100 °C for an additional 30 min. The resulting liquid was filtered, and its absorbance at 440 nm was measured. The gardenia–spice combination showing the highest absorbance was selected for subsequent experiments.

Based on these results, gardenia and licorice root were identified as the optimal combination and were co-boiled under the same conditions. One milliliter of the co-decocted liquid was collected every 30 min, and the concentrations of Croc and crocin in each sample were analyzed using high-performance liquid chromatography (HPLC), as described in Supporting Information S1. Gardenia boiled alone under identical conditions served as the control.

Subsequently, chicken pieces were immersed in both decoctions and simmered for 30 min until they were uniformly colored. Each chicken sample was visually inspected to assess the coloration effects of the decoctions.

### 2.3 Preparation and physicochemical characterization of Croc-Na_2_GA

Building upon previous studies on carotenoids (Focsan et al., 2019; Polyakov et al., 2009; Polyakov et al., 2023), this study employed a resonant acoustic mixing (RAM) approach to prepare the Croc-Na_2_GA supramolecular pigment. Croc and Na_2_GA were mixed at a 1:20 mass ratio in a 500 mL jar, and the mixture was processed using RAM at 30 Hz for 30 min. This process enabled the formation of a Croc-Na_2_GA complex with enhanced solubility and stability.

The physicochemical properties of Croc-Na_2_GA were assessed using multiple techniques. Scanning electron microscopy (SEM; Gemini 500, Zeiss, Germany) was employed to examine the solid-state morphology of the complex under an accelerating voltage of 5 kV. Differential scanning calorimetry (DSC) analysis was performed using a DSC-250 cell (TA Instruments, USA) under an argon atmosphere to evaluate thermal behavior. Functional groups in the samples were identified using Fourier transform infrared (FT-IR) spectroscopy. For analysis, dried samples were mixed with KBr, pressed into 1 mm pellets, and analyzed with an FTIR spectrometer (Spectrum Two, PerkinElmer, USA). Additionally, the crystalline and amorphous characteristics of the samples were examined through X-ray diffraction (XRD) analysis using an X-ray powder diffractometer (D2 PHASER, Bruker, Germany).

### 2.4 Stability and compatibility of Croc-Na_2_GA

The compatibility of Croc-Na_2_GA was first evaluated in the absence of common food additives, including glucose, sucrose, citric acid, xylitol, sodium benzoate, vitamin C, and maltose, as well as various metal ions such as Na^+^, K^+^, Ca^2+^, Cu^2+^, Fe^2+^, Al^3+^, Mg^2+^, Zn^2+^, and Ba^2+^. Solutions of these additives and metal ions were prepared at concentrations of 0.0012 mmol/L for food additives and 0.03 mmol/L for metal ions, following national guidelines (Alvarez & Rains, 1979; Fredrikson et al., 2002). Samples were mixed into these solutions until a color value of approximately 400 was achieved, and the mixtures were then stored in the dark at 25 ± 5 °C for 1 h before filtration. A full-wavelength scan was performed, and the absorbance at 440 nm was recorded. The change in color value at this wavelength, expressed as the percentage difference from the initial color value, was calculated to assess shifts in color intensity.

Second, the stability of Croc-Na_2_GA was investigated using Croc alone as the control. A drug high-light stability test chamber (SHH-GD-2, YSEI, China) was employed for light stability testing, with the color value at 400 nm serving as the evaluation parameter. Samples were placed in transparent tubes (2.5 cm in diameter) and exposed to LED light (4500 ± 500 lx) at 25 °C. The color values of the samples were recorded daily. For thermal stability analysis, Croc-Na_2_GA and the control gardenia yellow were dissolved in deionized water to obtain equivalent color values. The solutions were then heated in the dark at 30, 40, 50, 60, 70, 80, 90, or 100 °C for 1 h. The color values of the solutions were subsequently measured and compared. The pH stability test was conducted by dissolving Croc-Na_2_GA and gardenia yellow in deionized water and adjusting the pH levels to values within the range of 3 to 13 using NaOH and HCl solutions. All samples were diluted to equivalent color values and kept in the dark at 25 °C for 1 h prior to measurement.

Finally, an accelerated aging assessment was performed to evaluate long-term stability. Samples were sealed in airtight bags and stored in a drug stability test chamber (DRK672, Drick Instruments, China) under controlled conditions of 40 ± 2 °C and 75 ± 5% relative humidity for 6 months.

### 2.5 Coloring performance of Croc-Na_2_GA in meat products

Chicken meat was ground into a paste, and 0.01% (*w*/w) of Croc-Na_2_GA was added and thoroughly mixed, while 0.01% (w/w) Na_2_GA was used as the control. All mixtures were molded into circular patties measuring 10 × 10 cm^2^ and roasted at 230 °C for 20 min to produce the colored samples in patty form. The color-fixation capacities of both the colored and stamped samples were evaluated separately using a soaking method. Each sample was immersed in 100 mL of water, and soaking solutions were collected at 0, 3, 15, and 24 h to measure color values at 440 nm. After 24 h, color differences between pre- and post-soaking samples were analyzed. Chromaticity measurements were performed using a portable colorimeter (HP-2132, Shanghai Hanpu Optoelectronic Technology Co., Ltd., China), and color differences were determined based on L*, a*, and b* parameters. In addition, the color, aroma, and overall acceptability of the meat patties were evaluated by 10 randomly selected participants (Brera & Miraglia, 1996; Brown et al., 2016) of different ages and genders using a five-point descriptive scale. For comparative purposes, gardenia yellow was used as a control.

Subsequently, chicken breast pieces measuring 2 × 4 × 1 cm^3^ were cut and air-dried for 30 min to ensure adequate surface drying. A 10% pigment solution with a color value of approximately 400 was applied to the chicken breast surface using a stamping method. The stamped samples were then roasted at 230 °C for 20 min. It was expected that, if the pigments were prone to diffusion upon water exposure or to contamination by other substances during soaking, the clarity of the stamped patterns would gradually diminish.

To investigate this effect, the stamped samples were immersed in 100 mL of deionized water and stored at 15 ± 2 °C. The clarity of the stamped patterns was assessed at 0, 12, 24, and 48 h to determine the durability and color retention capacity of the pigmented samples during prolonged exposure to water.

### 2.6 Scavenging activity of Croc-Na_2_GA toward endogenous contaminants formed during heat treatment of meat products

The antioxidant activity of each sample was evaluated by measuring its ability to scavenge DPPH·, ABTS^+^·, hydroxyl, and superoxide anion radicals according to standard procedures, with vitamin C serving as the reference.

The effects of thermal processing and repeated heating cycles on the formation of 5-HMF and NDMA were also investigated. Chicken, squid, and cod were processed into meat patties following the procedure described in Section 2.7. Each sample was heated for 30 min once every 24 h, and after the final heating cycle, it was ground into a fine powder. The powder was then extracted with an 80% methanol solution at 30 °C for 30 min. The resulting extract was centrifuged at 8000 rpm for 10 min at 4 °C, and the supernatant was filtered through a 0.45 μm organic membrane to produce the sample solution. The concentrations of 5-HMF and NDMA were then quantified using HPLC analysis.

### 2.7 Coloring and color-fixation mechanisms of Croc-Na_2_GA

The mechanism underlying the strong coloration effect of Croc-Na_2_GA was investigated using the following method. A parallel artificial membrane permeability assay (PAMPA) was conducted to assess the permeation of the pigment across mucosal and cellular membranes. The assay was performed using 12-well Transwell plates equipped with polycarbonate membranes (pore size: 0.4 μm and surface area: 1.12 cm^2^), with Croc serving as the control. Each receiver compartment contained 1.5 mL of distilled water, while the donor compartment was filled with 0.5 mL of either Croc or Croc-Na_2_GA solution, corresponding to a Croc concentration of 1.2 mg/mL. The assembled system was incubated at 37 °C with agitation at 200 rpm for 180 min. At 30-min intervals (30, 60, 90, 120, 150, and 180 min), 1 mL aliquots were collected from the receiver compartment and replaced with fresh distilled water to maintain a constant volume. The collected samples were filtered through 0.22 μm membranes and analyzed using HPLC.

The penetration of Croc-Na_2_GA into the skin was examined using both pig skin and Strat-M synthetic membranes in a Franz vertical diffusion cell. Fresh pig skin was carefully depilated, with subcutaneous fat and connective tissues removed, and subsequently rinsed with normal saline. Phosphate-buffered saline (PBS, pH 7.4) was used as the receiving solution, with a total volume of 20 mL. Pigment samples were dissolved in PBS, and 1 mL of each solution was placed into the donor chamber. At 30-min intervals, 0.5 mL of the receiving solution was withdrawn and replaced with an equal volume of fresh PBS to maintain steady-state conditions. The Croc concentration in the receiving solution was determined using HPLC, and the cumulative release of the pigment over time was calculated.

The binding and fixation of Croc-Na_2_GA to muscle tissue were investigated using molecular simulation. Molecular docking was first performed between Croc and Na_2_GA to illustrate the self-assembly behavior. Subsequently, the most stable complex was docked with myoglobin and actin to explore the interaction mechanisms of Croc-Na_2_GA within meat systems. The 3D molecular structures of Croc and Na_2_GA were retrieved from the PubChem database, while the protein structures of myoglobin and actin were acquired from the RCSB PDB database. Molecular docking simulations were performed using AutoDock Vina (The Scripps Research Institute, USA).

In the initial stage, Na_2_GA was set as the receptor and Croc as the ligand. Binding cavities on Na_2_GA were selected following a full-coverage principle. The resulting complexes were ranked based on their binding energies, followed by analyses of intermolecular interactions and docking positions.

The Croc-Na_2_GA complex with the lowest binding energy was then selected as the ligand, while myoglobin, myosin, and actin were used as receptors to assess pigment–protein interactions individually. A network structure was constructed to evaluate interaction patterns within systems characterized by low binding energy. Finally, PyMOL was employed to visualize the geometric configurations of these interactions.

## 3 Results and discussion

### 3.1 Establishment of the Croc-Na_2_GA supramolecular pigment

**Fig. 1:**
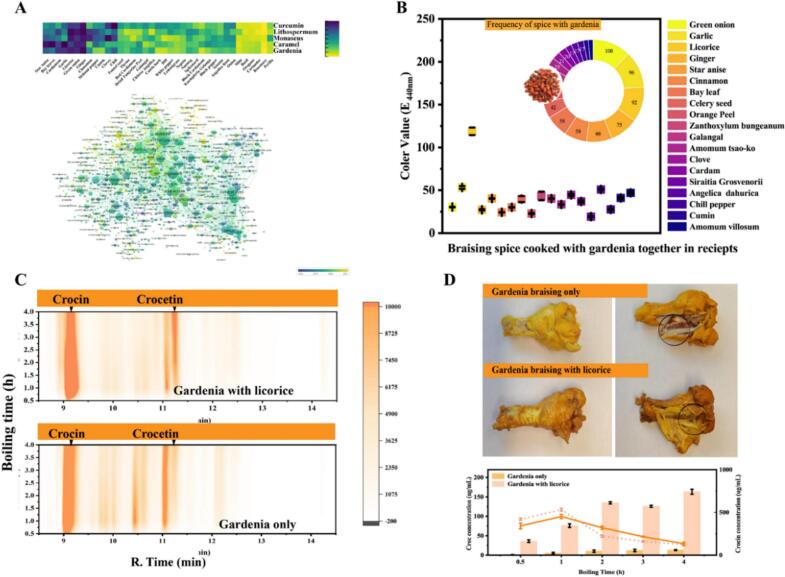
Development of the Croc-Na_2_GA supramolecular yellow pigment based on traditional Chinese recipes. (A) Network-based data mining of colorant compatibility in traditional Chinese braising recipes. (B) Composition of traditional gardenia-based braising formulations used to guide pigment development. (C) Changes in the chemical composition of the co-decoction (braising) liquid. (D) Coloring effects of gardenia and licorice, individually and in combination, on chicken meat, and the associated changes in Croc concentration during processing. (For interpretation of the references to color in this figure legend, the reader is referred to the web version of this article.)

**Supplementary Fig. S1:**
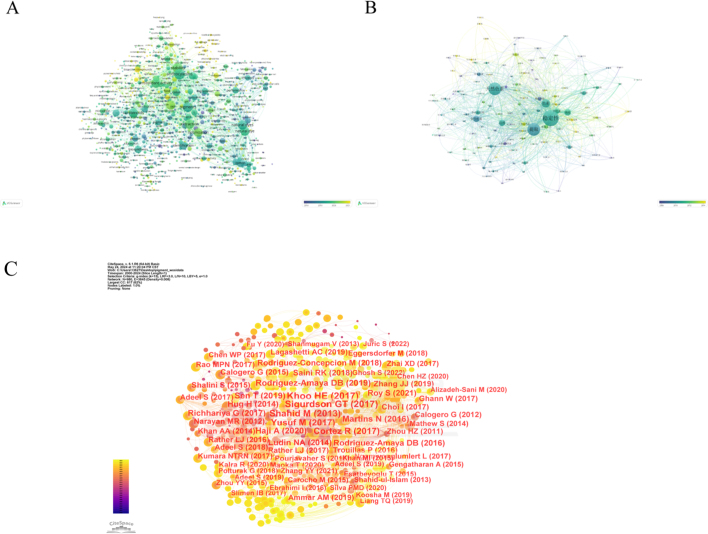
Keywords visual clustering map. (A) English keywords, (B) Chinese keywords and (C) Citation knowledge map in natural pigment related research.

Data mining indicated that gardenia, a traditional colorant widely used in meat preparation, was intentionally paired with spices in Chinese marinade recipes. Therefore, it was selected as the primary ingredient for exploring marinade combinations through network analysis. The findings (Figs. 1A and S1) revealed that, among more than 100,000 traditional Chinese braised meat recipes collected from online sources and literature, ingredients such as licorice, star anise, cinnamon, bay leaf, celery seed, orange peel, *Zanthoxylum bungeanum*, galangal, *Amomum tsao-ko*, clove, cardamom, *Siraitia grosvenorii*, *Angelica dahurica*, chili pepper, cumin, *Amomum villosum*, green onion, garlic, and ginger were frequently paired with gardenia, each exhibiting an incidence rate exceeding 5%. Moreover, the analysis indicated that a majority of traditional marinade recipes incorporated baking soda or sodium carbonate (with an occurrence rate above 75%) as non-spice ingredients to slightly raise the pH and facilitate meat tenderization. In summary, the compositional analysis of traditional gardenia-based marinades demonstrates that the formulation includes not only the previously mentioned spices but also a small quantity of an alkaline additive (such as baking soda) to enhance its effectiveness. Based on these findings, a scientific model integrating gardenia, spices, and a minimal amount of baking soda was developed in accordance with traditional marinade formulations for further experimental exploration.

Following the established framework, single-variable experiments were performed while maintaining gardenia and baking soda as the core components (Fig. 1B). Various spices were individually co-boiled with gardenia, and the color intensity of each decoction was assessed by measuring the color value at 440 nm (E_440_), with gardenia yellow serving as the reference. The results showed that the mixture of gardenia and licorice exhibited a significantly higher color value, approximately 2–3 times higher than those of other gardenia–spice mixtures. It was hypothesized that the combination of licorice and gardenia, in the presence of baking soda, enhances the extraction of yellow pigments from gardenia, thereby increasing the color value. This effect may be attributed to chemical reactions catalyzed by thermodynamic forces and to polymer self-assembly processes.

To further elucidate this mechanism, the chemical transformations occurring during co-decoction were analyzed. Compositional analysis revealed that significant chemical changes occurred during boiling (Fig. 1C). The crocin content decreased by 47%, while the Croc concentration in the extract of gardenia alone remained below 17 μg/g due to its poor water solubility. In contrast, when licorice was introduced, the Croc content increased significantly to 165.37 μg/g, while the crocin content decreased by 69%.

Coloring performance evaluation was subsequently conducted using chicken (representing white meat) treated with the co-boiled gardenia–licorice mixture (Fig. 1D). When treated with the extract of gardenia alone, the meat surface exhibited a pale yellowish-green hue, and its interior remained visibly white. In contrast, the application of the gardenia–licorice mixture produced a more vibrant and visually appealing reddish tone. The pigment penetrated deeply into the meat and created an appearance similar to that of traditionally stewed meat. These results demonstrate that the combination of gardenia and licorice enhances both the color intensity and the depth of pigment infusion in meat.

According to a previous report (Inoue et al., 2014), crocin found in gardenia is an ester glycoside that readily undergoes hydrolysis under alkaline conditions. The boiling of licorice generates an alkaline solution that facilitates the conversion of weakly hydrophilic GA into its more soluble salt form. Glycyrrhizin acts as a natural surfactant with hydrophilic properties, enabling it to form supramolecular aggregates with otherwise insoluble substances in the solution. Consequently, it can be inferred that in this system, Croc (derived from crocin in gardenia) and GA salt (derived from glycyrrhizin in licorice) self-assemble into supramolecular structures. This self-assembly enhances Croc solubility, leading to supersolubility. Moreover, these supramolecular aggregates exhibit improved biocompatibility, facilitating their penetration into subcutaneous tissues and thereby enhancing coloration efficiency.

In summary, Croc extracted from gardenia and Na_2_GA derived from licorice were identified as effective yellow pigment components under alkaline conditions, demonstrating superior coloring performance for this formulation.

### 3.2 Preparation and physicochemical characterization of Croc-Na_2_GA

**Fig. 2:**
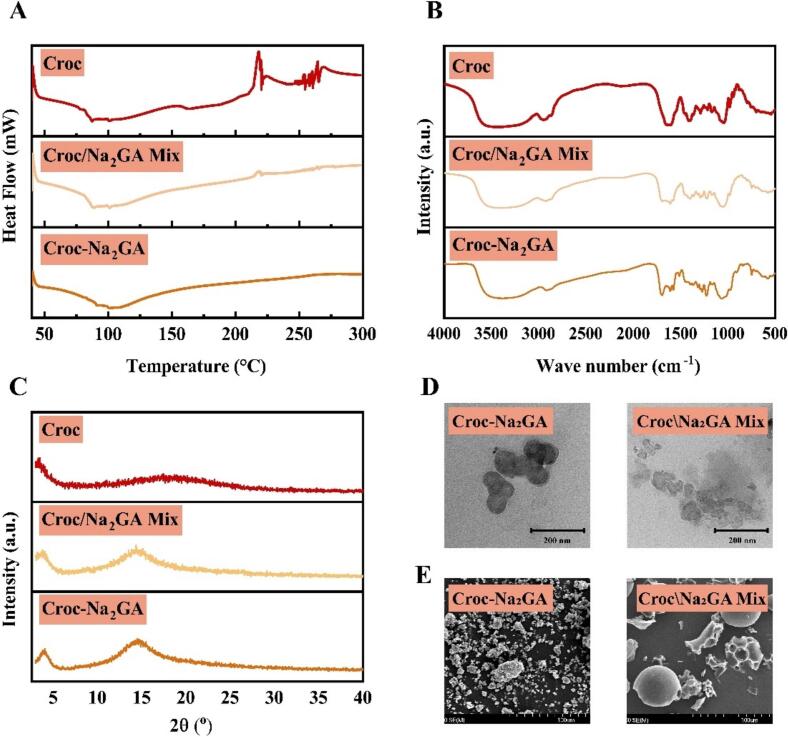
Structural characterization and microstructural analysis of Croc-Na_2_GA (Croc as the control). (A) The DSC spectrum, (B) FTIR spectrum, (C) XRD spectrum, (D) TEM image, and (E) SEM image of the supramolecular pigment and physical mixture.

Based on the results of the preceding study, Croc was employed as the yellow pigment, while Na_2_GA served as the carrier. A RAM solid-phase technique (details provided in Section 2 of the Supporting Information) was applied to prepare the Croc-Na_2_GA supramolecular pigment, aiming to reduce preparation losses. Subsequent XRD, DSC, and FT-IR investigations (Fig. 2A–C) confirmed the formation of a supramolecular complex, with detailed analyses provided in Section 3 of the Supporting Information.

The morphology, structural characteristics, and self-assembly behavior of Croc-Na_2_GA in aqueous solution were evaluated using SEM, TEM, and particle size analysis. SEM analysis revealed that the physical mixture of Croc and Na_2_GA exhibited distinct crystalline and spherical morphologies. In contrast, the Croc-Na_2_GA supramolecular system displayed smaller, irregularly shaped particles. TEM analysis showed that amorphous Croc and Na_2_GA self-assembled into well-defined spherical micelles in aqueous environments. These micelles exhibited smooth surfaces and clear internal boundaries, suggesting the formation of nanocapsules capable of maintaining stability under physiological conditions. SEM observations (Fig. 2E) confirmed that the combination of Croc and Na_2_GA produced an amorphous structure in the Croc-Na_2_GA complex. Compared to Croc, Na_2_GA, and their physical mixture, the Croc-Na_2_GA solid dispersion displayed a disordered amorphous morphology with reduced particle size, thereby enhancing the specific surface area and solubility of the pigment. To assess the dispersion behavior of Croc within the Croc-Na_2_GA system, nanoparticle size and zeta potential measurements were conducted. Unprocessed Croc showed poor aqueous dispersion, forming large, irregular particles. In contrast, the Croc-Na_2_GA solution displayed well-dispersed nanoparticles with an average size of 197.30 nm and a highly negative zeta potential of −94.90 mV, suggesting that the Croc-Na_2_GA formulation produced stable, negatively charged nanoparticles with a uniform size distribution. The enhanced dispersibility and colloidal stability of Croc in this formulation likely improved its solubilization and bioavailability, consistent with previous findings (Liao et al., 2024; Su et al., 2023; Xu et al., 2021). Overall, these findings confirm that Croc is effectively integrated into Na_2_GA in an amorphous form, substantially enhancing its water solubility and facilitating intestinal absorption, thereby increasing its permeability.

### 3.3 Stability and compatibility of Croc-Na_2_GA

**Fig. 3:**
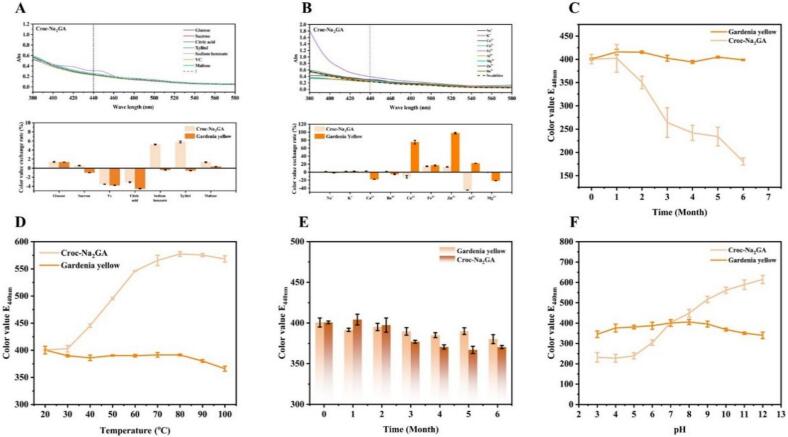
Stability evaluation of Croc-Na_2_GA (gardenia yellow as the control). Assessment of the pigment stability under different conditions: (A) in the presence of common food additives, (B) in the presence of various metal ions, (C) under strong light exposure, (D) at different temperatures, (E) under an accelerated aging test, and (F) in solutions with different pH values. (For interpretation of the references to color in this figure legend, the reader is referred to the web version of this article.)

To evaluate the potential application of Croc-Na_2_GA in food production, a preliminary study was conducted to examine how common food additives and metal ions influenced its maximum absorption wavelength (indicating color) and color value (reflecting color richness), with gardenia yellow serving as the control. Common food additives had no significant effect on either the maximum absorption wavelength or color value of gardenia yellow or Croc-Na_2_GA (Fig. 3A). Further analysis showed that acidic additives slightly reduced the color intensity of Croc-Na_2_GA, whereas alkaline additives improved its color vibrancy. Nonetheless, all variations remained within a 10% range, indicating that Croc-Na_2_GA is compatible with commonly used food additives and exhibits performance comparable to that of gardenia yellow.

The effects of various metal ions on the hue and color intensity of gardenia yellow and Croc-Na_2_GA were also investigated **(**Fig. 3B**)**. Because gardenia yellow is prone to oxidation and complex formation, metal ions with oxidizing or complexing properties significantly affected its coloration (Ácsová et al., 2022). Specifically, Cu^2+^, Fe^3+^, and Zn^2+^ deepened the color, while Al^3+^ and Mg^2+^ produced lighter tones due to complexation and adsorption effects. In contrast, these metal ions had a minimal impact on the hue and color intensity of Croc-Na_2_GA. This enhanced stability can be attributed to the highly stable micellar structures formed by Croc and Na_2_GA within the supramolecular complex, where Na_2_GA forms a hydrophilic shell that encapsulates Croc. Additionally, Na_2_GA possesses strong ionic stability, resistance to precipitation and complexation, and an ability to scavenge free radicals, thereby protecting Croc from degradation (Ageeva et al., 2022). Consequently, Croc-Na_2_GA demonstrates reduced sensitivity to metal ions and superior application potential compared to Croc alone.

The photosensitivity and storage stability of Croc-Na_2_GA are illustrated in Fig. 3C. Compared to gardenia yellow, Croc-Na_2_GA showed no notable color deterioration during storage in the dark. After 1 week of dark storage, both colorants exhibited <10% color loss, indicating the excellent stability of Croc-Na_2_GA in sealed, light-protected environments. However, upon light exposure, the stability of Croc-Na_2_GA decreased significantly, while gardenia yellow remained relatively stable. This stability reduction may be attributed to radical propagation within the Na_2_GA matrix under illumination, which promotes interactions with Croc molecules, making them more susceptible to degradation and thus lowering overall stability. Therefore, Croc-Na_2_GA is best suited for storage and use under dark conditions. Since colored meat products are generally wrapped in aluminum foil and protected from prolonged light exposure, the limited photostability of Croc-Na_2_GA does not significantly affect its practical applications.

The thermal stability of Croc-Na_2_GA is presented in Fig. 3D. Within the temperature range of 20–80 °C, both Croc and Croc-Na_2_GA maintained relatively stable color values. Above 80 °C, gardenia yellow underwent rapid decomposition, leading to significant darkening and color loss. In contrast, the coloring performance of Croc-Na_2_GA increased steadily with temperature, reaching its maximum color intensity at 80 °C. These findings indicate that Croc-Na_2_GA possesses enhanced thermal stability and superior coloring performance at elevated temperatures, making it more suitable than gardenia yellow for thermal food processing applications.

To assess the long-term stability of Croc-Na_2_GA and determine its optimal storage conditions, an accelerated aging test was conducted for 6 months following the standard protocol (GB 7718–2025) (González-González et al., 2022). After storage at 40 ± 2 °C and 75 ± 5% relative humidity for 6 months, Croc-Na_2_GA demonstrated stability comparable to that of gardenia yellow (Fig. 3E). The complex retained its yellow hue and loose powder form, with color values exceeding 425. These findings confirm that Croc-Na_2_GA maintains good stability under various conditions, except when exposed to light or acidic environments. Thus, it is best suited for use in neutral to alkaline conditions and should be stored away from light. Since the processing of fresh meat products typically occurs within 1–2 days, the light stability of Croc-Na_2_GA is adequate for practical applications.

The influence of pH on the stability of Croc-Na_2_GA is illustrated in Fig. 3F. Native Croc exhibits poor solubility in water across the pH range relevant to food systems, with only slight variations in color value. Conversely, in Croc-Na_2_GA, acidic conditions (pH 3–6) reduced the solubility of the GA carrier, leading to micellar precipitation and a slight reduction in color value. Under neutral to alkaline conditions (pH 8–12), Na_2_GA primarily existed in an anionic form, increasing its hydrophilicity and facilitating the formation of stable, water-dispersible complex micelles that significantly increased the color value. This behavior suggests that the water-soluble complex locally precipitates upon contact with the slightly acidic environment of muscle tissues, forming microcapsule-like structures within the tissue matrix. This process enhances color retention and reduces pigment leaching, enabling effective and durable coloration.

### 3.4 Coloring performance and fixation efficiency of Croc-Na_2_GA in roasted meat products

**Fig. 4:**
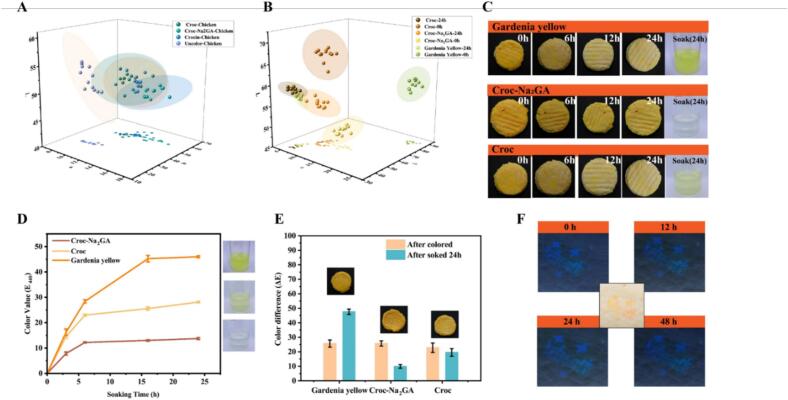
Coloring performance of Croc-Na_2_GA in thermally processed meat (gardenia yellow and Croc as the controls). (A) Chroma values of roasted chicken samples treated with different natural yellow pigments. (B) Chroma changes before and after soaking. (C) Appearance changes at different soaking time. (D) Color values of soaking solutions at different soaking times. (E) Appearance and chroma changes after 24 h of soaking. (F) Patterns stamped on thermally processed meat using Croc-Na_2_GA and their clarity changes during soaking (negative images applied to improve visualization). (For interpretation of the references to color in this figure legend, the reader is referred to the web version of this article.)

An investigation was conducted to evaluate the coloring performance of Croc-Na_2_GA on chicken patties. Chromaticity measurements were employed to analyze the effects of Croc-Na_2_GA, Croc, and gardenia yellow on meat coloration. The results are presented in Fig. 4A. In chromaticity analysis, the *L* value represents brightness or darkness, while the *a* and *b* values denote the red–green and yellow–blue dimensions, respectively (Tuberoso et al., 2014). Uncolored patties exhibited uneven coloration, displaying a reddish and dull appearance that differed markedly from the desired yellow hue. The application of Croc, gardenia yellow, and Croc-Na_2_GA produced comparable coloration outcomes, with each yielding a vibrant, warm yellow hue. However, the shade produced by Croc-Na_2_GA appeared slightly more reddish and warmer, potentially enhancing visual appeal and stimulating appetite.

Subsequent investigations assessed color retention in chicken patties treated with the three pigments after soaking. Chromaticity measurements were performed before and after 24 h of soaking, and the results are presented in Fig. 4B and C, respectively. Samples colored with gardenia yellow and Croc (serving as controls) exhibited notable color fading after soaking. The soaking solutions turned bright yellow, while the patties became significantly lighter, evidenced by increased *L* values (indicating higher brightness) and reduced *a* and *b* values (indicating a transition from bright yellow to a paler tone). In contrast, patties colored with Croc-Na_2_GA showed minimal color loss, with the soaking solution remaining nearly colorless and the patties retaining their original color after 24 h.

To further investigate the degree of pigment leaching, the color value of the soaking solution was assessed at three-hour intervals throughout the soaking process, and the variations in color values before and after soaking were analyzed (Fig. 4D and E). The results demonstrated that patties treated with Croc-Na_2_GA exhibited superior color stability compared to those treated with Croc or gardenia yellow. Given the pronounced resistance of Croc-Na_2_GA to color fading, its ability to prevent staining was further explored.

The staining resistance of Croc-Na_2_GA during soaking was subsequently analyzed **(**Fig. 4F**)**. Any reduction in clarity or the appearance of blurriness indicated pigment diffusion through water, leading to discoloration in previously unstained areas. To enhance visual contrast, images were captured using a negative film setting. The findings demonstrated that chicken breast samples treated with Croc-Na_2_GA maintained sharp and well-defined patterns even after prolonged soaking. After 48 h, the pattern remained clearly visible, indicating that Croc-Na_2_GA possesses excellent color retention and fixation capability.

Finally, a sensory evaluation was performed to evaluate the coloring performance of the three pigments (Table 1**)**. Chicken patties were colored with Croc-Na_2_GA, gardenia yellow, or Croc. All colored patties retained a natural meat aroma without any undesirable odors. No significant differences were observed in color, aroma, or overall preference between samples treated with Croc-Na_2_GA and those colored with gardenia yellow, indicating that Croc-Na_2_GA is comparably effective to gardenia yellow for meat coloration. However, patties colored with Croc received slightly lower color scores compared to the other two groups. These results demonstrate that Croc-Na_2_GA serves as an effective alternative to gardenia yellow for food coloring, providing stable coloration while maintaining sensory qualities.

**Table 1:** Sensory scores of meat samples treated with different colorants.

Organoleptic standard	Croc-Na_2_GA	Gardenia Yellow	Croc
Color	4.90 ± 0.31^a^	4.75 ± 0.44^a^	4.40 ± 0.50^b^
Aroma	4.55 ± 0.51^a^	4.60 ± 0.50^a^	4.50 ± 0.51^a^
Overall acceptability	4.75 ± 0.44^a^	4.75 ± 0.44^a^	4.45 ± 0.51^a^

Different letters indicate significant differences within each row (*P* < 0.05).

### 3.5 Coloring and color-fixation mechanisms of Croc-Na_2_GA

**Fig. 5:**
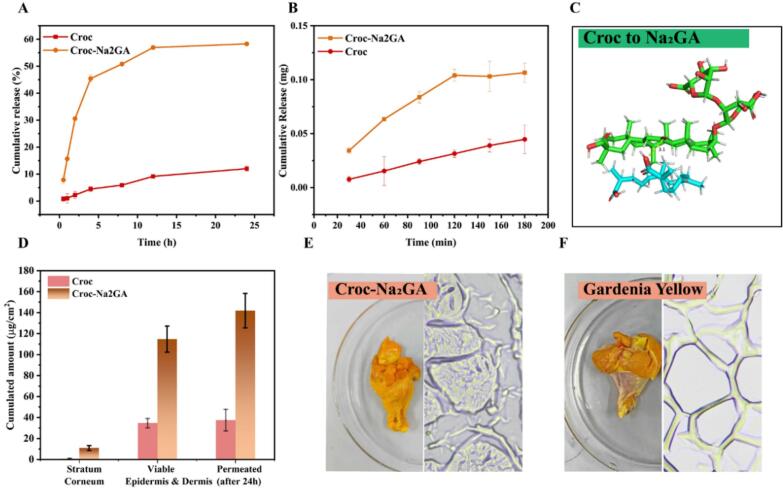
Dissolution profiles and tissue permeability of Croc-Na_2_GA. (A) Dissolution behavior of Croc-Na_2_GA (Croc as the control). (B) PAMPA-based permeability assessment (Croc as the control). (C) A molecular docking model for Na_2_GA and Croc. (D) Permeability through pig skin (Croc as the control). (E) Permeability in chicken tissue and (F) the associated permeability comparison with gardenia yellow. (For interpretation of the references to color in this figure legend, the reader is referred to the web version of this article.)

The enhanced color stability of Croc-Na_2_GA can be attributed to the formation of micelles with efficient release properties and improved tissue absorption. A molecular docking study involving Croc and Na_2_GA was conducted, and the results are presented in Fig. 5C. The calculated binding energy for the interaction between Croc (depicted in blue) and Na_2_GA (shown in green) was −3.834 kcal/mol, indicating a strong interaction primarily driven by hydrogen bonding. Structural analysis further revealed that hydrophobic interactions between the multi-alkyl ring of Na_2_GA and the alkyl chain of Croc enhanced the stability of the supramolecular assembly.

These findings suggest that Croc-Na_2_GA forms a self-assembled solid dispersion capable of generating uniform, nanosized micelles in aqueous solution. Croc disperses rapidly in high-concentration aqueous solutions. Consistent with previous findings (Apanasenko et al., 2015; Zhu et al., 2022), these nanomicelles demonstrate favorable membrane permeability, facilitating the transport of poorly water-soluble compounds. Accordingly, the proposed coloring mechanism indicates that these nanomicelles penetrate tissue cells more effectively, where they bind to muscle proteins and release Croc, forming fine and stable pigment precipitates that enhance staining and color-fixation efficiencies.

Preliminary dissolution tests supported this hypothesis, showing that Croc-Na_2_GA exhibited a markedly higher in vitro dissolution rate compared to Croc (Fig. 5A). Its dissolution rate exceeded 60% within 5 min and surpassed 90% after 90 min, while Croc displayed a dissolution rate below 20%. This indicates that Croc-Na_2_GA dissolves more effectively in water, producing a concentrated solution beneficial for its coloring performance. Subsequent in vitro permeation tests using an artificial membrane (Fig. 5B) showed that the permeability of Croc-Na_2_GA was nearly five times greater than that of Croc**.** To further assess the tissue penetration ability of the pigment, natural pig skin and chicken meat were employed as model tissues. The permeability test using pig skin **(**Fig. 5D) demonstrated that Croc-Na_2_GA rapidly penetrated the epidermis and accumulated in the dermal and subcutaneous layers. In these regions, the concentrations of Croc-Na_2_GA were 3–5 times higher than those of Croc, highlighting the significantly improved penetration capability of Croc-Na_2_GA.

Additional immersion experiments (Fig. 5E and F) revealed that chicken samples soaked in Croc-Na_2_GA solution exhibited thorough pigment penetration and intense coloration. Microscopic observation conducted upon fixation of tissue sections revealed that nearly all cells within and between tissues exhibited a yellow hue following Croc-Na_2_GA treatment. In contrast, in samples soaked in gardenia yellow solution, staining was limited to intercellular spaces, leaving most cells uncolored. These findings indicate that Croc-Na_2_GA significantly enhances pigment permeation, allowing the colorant to reach cell interiors and achieve a more pronounced staining effect compared to gardenia yellow.

**Fig. 6:**
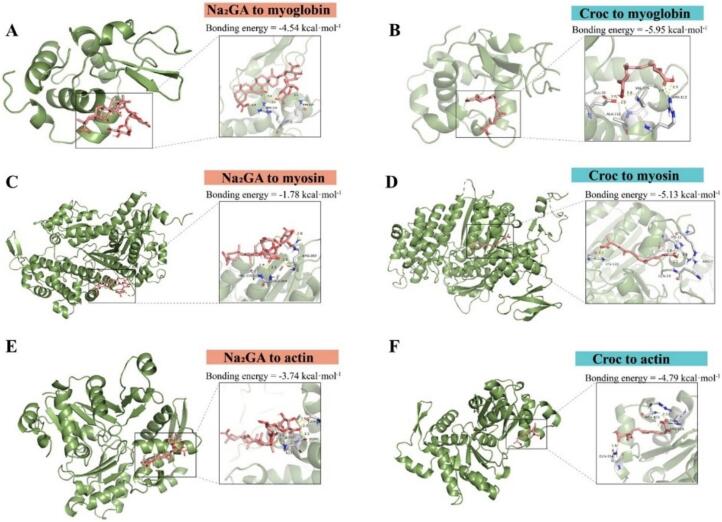
Molecular docking analysis of Na_2_GA and Croc with major muscle proteins. Interactions of Na_2_GA and Croc with (A–B) myoglobin, (C–D) myosin, and (E–F) actin.

To further investigate the mechanism behind color fixation, molecular docking was performed to examine the interactions between Croc-Na_2_GA and major muscle proteins. The supramolecular complex was docked individually with myoglobin (2Q0M, Fig. 6A), myosin (1VOM, Fig. 6C), and actin (1J6Z, Fig. 6E). The corresponding binding energies were − 4.54 kcal/mol, −1.78 kcal/mol, and − 3.74 kcal/mol, respectively, with hydrogen bonding identified as the primary interaction mode. These moderate binding affinities suggest that Croc-Na_2_GA does not form highly stable complexes with these proteins.

Based on these findings, it was hypothesized that the supramolecular pigment initially diffuses through the tissue matrix and subsequently interacts with myoglobin, actin, and myosin, thereby enhancing pigment retention and fixation within the meat. To assess the binding behavior of Croc, molecular docking studies were performed between Croc and the same three muscle proteins. Croc demonstrated the strongest interaction with myoglobin (2Q0M), with a binding energy of −5.95 kcal/mol (Fig. 6B). The dicarboxyl group of Croc formed hydrogen bonds with residues GLU-35, ALA-110, VAL-109, and ARG-112, all with bond lengths below 3.0 Å, indicating strong binding affinity. Similarly, Croc displayed a favorable interaction with myosin (1VOM), yielding a binding energy of −5.13 kcal/mol (Fig. 6D), forming hydrogen bonds with residues LYS-130, HIS-12, ARG-7, and GLN-19, all within the optimal bond length (< 3.0 Å). For actin (1J6Z), the binding energy was −4.79 kcal/mol (Fig. 6F), involving hydrogen bonds with ARG-116, ARG-372, and GLN-354.

The docking analysis indicates that, compared to Croc-Na_2_GA, Croc has a significantly stronger binding affinity for muscle proteins. This suggests that upon entering tissue, Croc-Na_2_GA releases Croc, which then binds tightly to these proteins, leading to effective pigment immobilization within the tissue. These interactions contribute to the excellent color-fixation capability of Croc-Na_2_GA. Overall, these findings reveal that the effective coloring and color-fixation mechanisms of Croc-Na_2_GA involve several interrelated processes. Initially, Croc and Na_2_GA form highly soluble nanomicelles in aqueous solution. These micelles possess excellent membrane permeability, allowing Croc to penetrate deeply into tissues while minimizing pigment accumulation on the tissue surface. Upon reaching the tissue, the micelles disintegrate and release Croc, which possesses strong protein-binding capacity and low water solubility. Accordingly, Croc accumulates within the tissue as small and stable color lakes, thereby improving the coloring efficiency and color-fixation performance of Croc-Na_2_GA.

### 3.6 Scavenging activity of Croc-Na_2_GA toward endogenous contaminants formed during heat treatment

This study further evaluated the ability of Croc-Na_2_GA to scavenge free radicals and inhibit the formation of endogenous contaminants during thermal processing, including harmful chemicals, 5-HMF, and NDMA.

**Fig. 7:**
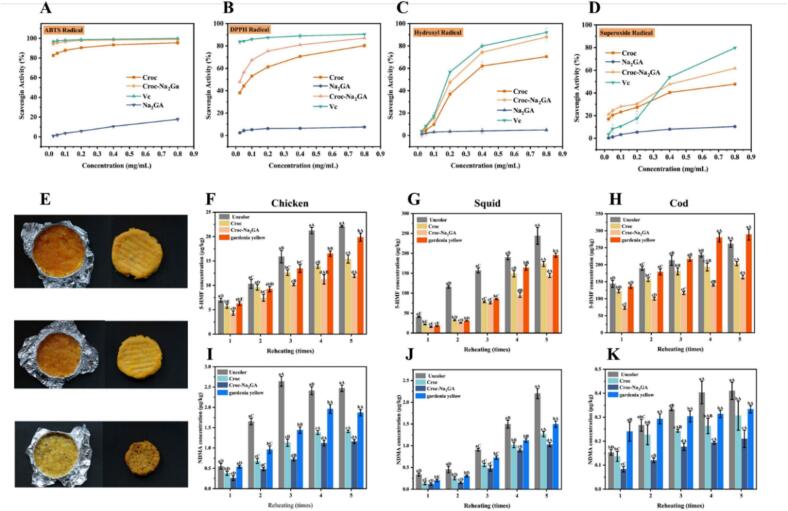
Antioxidant activity and contaminant-reducing effects of Croc-Na_2_GA. (A–D) Scavenging activities of Croc-Na_2_GA against (A) ABTS^+^·, (B) DPPH·, (C) hydroxyl, and (D) superoxide radicals in solution (Croc as the control). (E) Appearances of meat samples before and after cooking. (F–H) Concentrations of 5-HMF in (F) chicken, (G) squid, and (H) cod after different reheating cycles. (I–K) Concentrations of NDMA in (I) chicken, (J) squid, and (K) cod after different reheating cycles.

The antioxidant properties of Croc-Na_2_GA were examined through several free radical scavenging assays, including DPPH·, ABTS^+^·, hydroxyl radicals, and superoxide anion radicals (Fig. 7A–D). Na_2_GA alone demonstrated scavenging rates below 20% for all four radicals, indicating limited antioxidant capacity and confirming that it was not the main radical scavenger in the system. Compared to Croc, Croc-Na_2_GA showed significantly higher scavenging activity against all four radicals (Fig. 7). Within the tested concentration range, both Croc and Croc-Na_2_GA achieved scavenging rates above 80% for ABTS^+^·. The half-maximal inhibitory concentration (IC_50_) values for Croc-Na_2_GA were 0.026 mg/mL for DPPH·, 0.210 mg/mL for hydroxyl radicals, and 0.373 mg/mL for superoxide anion radicals. These results indicate that Croc-Na_2_GA possesses strong radical-scavenging capability and pronounced antioxidant activity. Moreover, this enhanced antioxidant capacity cannot be attributed solely to Na_2_GA, suggesting a synergistic interaction between Croc and Na_2_GA. Specifically, the incorporation of Na_2_GA promotes the self-assembly of Croc into nanomicellar structures, a process that appears to enhance antioxidant properties through these synergistic molecular interactions.

NDMA and 5-HMF are key endogenous contaminants associated with nitrosamines and furfurals, respectively. These compounds are potential carcinogens that readily form and accumulate during the thermal processing of meats and the repeated reheating of various foods (Song et al., 2015). Therefore, preventing their formation is crucial for ensuring food safety. Previous studies have indicated that NDMA and 5-HMF are primarily produced via lipid peroxidation and the Maillard reaction (Noh et al., 2024), both of which can be effectively inhibited by radical scavengers. Given the strong radical-scavenging properties of Croc-Na_2_GA, this study evaluated its ability to inhibit the formation of NDMA and 5-HMF during the thermal processing of food. To improve the relevance of the findings, chicken, cod, and squid were selected as experimental materials, since fish and mollusks are more prone to produce these contaminants compared to white meat (Xu et al., 2015). The results are presented in Fig. 7F–K. In the control group (without additives), 5-HMF concentrations were highest in cod (261.53 ± 9.99 μg/kg), followed by squid (243.86 ± 21.27 μg/kg) and chicken (22.11 ± 0.18 μg/kg), confirming the greater susceptibility of fish to carcinogen formation during repeated heating. The addition of Croc and gardenia yellow resulted in moderate (∼5%) reductions in 5-HMF and NDMA formation in reheated samples. In contrast, the incorporation of Croc-Na_2_GA resulted in nearly a 50% reduction in both 5-HMF and NDMA concentrations across all tested samples (chicken, cod, and squid). Notably, after five reheating cycles, the concentrations of these contaminants in the Croc-Na_2_GA group were significantly lower than those in the other groups.

In summary, Croc-Na_2_GA exhibits strong free radical scavenging capability and effectively reduces the formation of harmful compounds such as 5-HMF and NDMA during repeated heating of meat products. These results demonstrate that Croc-Na_2_GA serves not only as an effective natural pigment but also as a protective additive that reduces the production of harmful substances during thermal processing. It thus meets the demands of modern food processing, particularly in the production of pre-cooked or ready-to-eat meals. By ensuring stable and safe coloration while minimizing the risks associated with repeated heating, Croc-Na_2_GA shows considerable promise for application in the food industry.

Despite these promising findings, several limitations should be acknowledged. First, the photostability of Croc-Na_2_GA remains insufficient for applications involving prolonged light exposure, as evidenced by the accelerated color degradation observed under continuous LED illumination (4500 ± 500 lx). Although this limitation may restrict its use in unpackaged retail display environments, it is unlikely to compromise applications involving vacuum-packaged or aluminum-foil-wrapped meat products. Second, the optimal pH stability range of Croc-Na_2_GA is confined to neutral-to-alkaline conditions (pH 8–13), with reduced performance under acidic conditions due to micellar precipitation. This characteristic may limit its applicability in acidic meat formulations, including certain marinated and fermented products. Future studies should therefore focus on surface-modification strategies or secondary encapsulation approaches to broaden the operational pH range and improve photostability. In addition, although the molecular docking analyses provided valuable mechanistic insights, experimental validation using binding techniques such as surface plasmon resonance or isothermal titration calorimetry would further strengthen the proposed mechanism. Long-term in vivo toxicity assessments and large-scale consumer sensory evaluations are also necessary to support future commercialization of this supramolecular pigment system.

## 4 Conclusion

In conclusion, this study developed Croc-Na_2_GA, a non-covalent supramolecular complex derived from traditional Chinese recipes and specifically designed for meat-coloring applications, to address the safety, stability, and regulatory challenges associated with natural pigments.

Croc-Na_2_GA exhibited high water solubility (158.37 ± 1.06 mg/100 mL) and enhanced stability under varying temperatures, strong light exposure, a wide pH range, and the presence of common food additives and metal ions. Compared to commercially available gardenia yellow, it showed a 15–40% improvement in stability without noticeable color degradation. Coloring performance tests further demonstrated that Croc-Na_2_GA nanomicelles possessed superior permeability through skin and mucosal tissues and displayed strong affinity for muscle proteins (myoglobin, actin, and myosin), resulting in significantly improved color depth and fastness relative to gardenia yellow, supporting consumer acceptance. Additionally, Croc-Na_2_GA effectively scavenged 5-HMF and NDMA during repeated heating. Notably, in processed meat products—including white meat, fish, and seafood—the concentrations of these substances decreased by up to 50% after five reheating cycles, compared to those observed in control samples. These findings indicate that Croc-Na_2_GA functions not only as a stable and efficient colorant but also as an active agent capable of suppressing the generation of hazardous compounds during thermal processing. Moreover, since its constituents are derived from traditional food additives, the formulation offers strong potential for scalability and industrial application.

Collectively, this study on supramolecular pigments highlights the profound scientific principles embedded in traditional Chinese culinary practices and provides valuable guidance for developing high-performance, clean-label food additives. Furthermore, this work establishes a novel paradigm for the scientific investigation of traditional dietary formulations and the rational design of next-generation food additives, offering significant implications for advancing the food industry and fostering innovation in functional food ingredients.

The following are the supplementary data related to this article.

**Supplementary Fig. S2:**
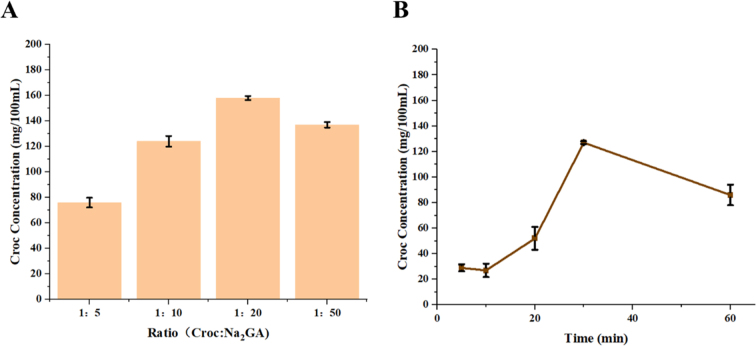
Solubility of crocetin under varying formulation parameters: (A) Croc:Na_2_GA mass ratio; (B) RAM treating time.

**Supplementary Fig. S3:**
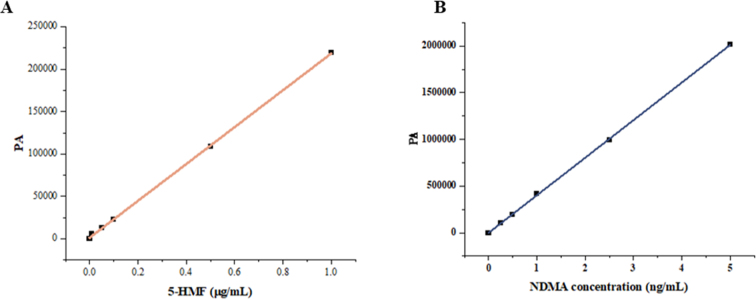
Calibration curves of 5-HMF(A) and NDMA(B).

## CRediT authorship contribution statement

**Qinxue Ni:** Writing – review & editing, Writing – original draft, Validation, Methodology, Conceptualization. **Haici Lan:** Resources, Formal analysis, Data curation. **Shuang Chen:** Writing – original draft, Formal analysis, Data curation. **Xiaolong Zhang:** Methodology. **Yanming Ren:** Funding acquisition, Data curation. **Wenhao Xu:** Resources, Methodology, Formal analysis, Data curation. **Youzuo Zhang:** Formal analysis, Data curation.

## Consent statement

All human subjects involved in the sensory evaluation were informed prior to participation.

## Declaration of competing interest

The authors declare that they have no known competing financial interests or personal relationships that could have appeared to influence the work reported in this paper.

## Data Availability

Data will be made available on request.
